# Clinically suspected T4 colorectal cancer may be resected using a laparoscopic approach

**DOI:** 10.1186/s12885-016-2753-8

**Published:** 2016-09-05

**Authors:** Jong Seob Park, Jung Wook Huh, Yoon Ah Park, Yong Beom Cho, Seong Hyeon Yun, Hee Cheol Kim, Woo Yong Lee, Ho-Kyung Chun

**Affiliations:** 1 Department of Surgery, CHA Gangnam Medical Center, CHA University School of Medicine, Seoul, South Korea; 2 Department of Surgery, Samsung Medical Center, Sungkyunkwan University School of Medicine, 81 Irwon-ro, Gangnam-gu, Seoul 135-710 South Korea; 3 Department of Surgery, Kangbuk Samsung Hospital, Sungkyunkwan University School of Medicine, Seoul, South Korea

**Keywords:** Clinical T4, Colorectal cancer, Laparoscopy, Oncologic outcome

## Abstract

**Background:**

The role of laparoscopic resection in patients with clinically suspicious T4 colorectal cancer remains controversial. The aim of this study was to compare the long-term and oncologic outcomes of laparoscopic resection and the open approach in clinical T4 colorectal cancer.

**Methods:**

Two hundred ninety-three consecutive patients undergoing curative surgery for colorectal cancer suspected to be T4 by computed tomography and/or magnetic resonance imaging were reviewed.

**Results:**

Despite clinical suspicion of T4 disease in all cases, concordance with pathologic determination of T4 was only 37.9 %. Of the 71 patients in the laparoscopic group, four (5.6 %) were converted to the open technique. Patients in the laparoscopic group had significantly lower estimated blood loss (*p* < 0.001), fewer days to first flatus (*p* = 0.001), shorter length of hospital stay (*p* < 0.001), and fewer adverse events (14.1 % versus 31.5 %, *p* = 0.004). After a median follow-up of 36 months, 5-year disease-free survival was not significantly different between the two groups (81.8 % in laparoscopic versus 73.9 % in open surgery, *p* = 0.433). The clinical factors that predicted T4 staging on pathologic examination were found to be male sex (*p* = 0.038), preoperative carcinoembryonic antigen status (*p* = 0.021), clinical N status (*p* = 0.046), and clinical cancer perforation (*p* = 0.004).

**Conclusions:**

Laparoscopic colorectal resection for T4 colorectal cancer has perioperative and long-term oncologic outcomes similar to those of the open approach when performed by an experienced surgeon.

## Background

Laparoscopic surgery is a well-established treatment approach for colorectal cancer. Several randomized studies have reported that the laparoscopic approach is associated with decreased postoperative pain, shorter hospital stay, and reduced postoperative adverse events compared to conventional surgery [1–4]. Recently, the guidelines from the American Society of Colon & Rectal Surgeons (ASCRS) and the European Association of Endoscopic Surgery have suggested that a laparoscopic approach is the optimal technique for colorectal cancer resection [5–7].

However, the role of laparoscopic resection in patients with clinically suspected T4 colorectal cancer remains controversial. The concerns regarding laparoscopy at this stage of disease include higher risk of conversion and lower quality of oncologic resection. It is recommended that resection for locally advanced colorectal cancer be performed via an open approach, according to the Society of American Gastrointestinal and Endoscopic Surgeons guidelines (SAGES); however, the ASCRS guidelines suggest that laparoscopic and open colectomies result in equivalent oncological outcomes for localized colon cancer [5, 7].

There are several studies showing that a laparoscopic approach in locally-advanced colorectal cancer is a feasible and effective treatment option, but little information is currently available [8–13]. Thus, the aim of this study was to compare the long-term and oncologic outcomes of laparoscopic resection and open approach for clinically suspected T4 colorectal cancer.

## Methods

Patients who underwent colorectal cancer surgery from January 2000 to December 2010 were analyzed. Patients with pathologically confirmed primary colorectal cancer who underwent curative resection and had clinically suspicious T4 disease were included in this study. Patients with no recorded clinical T stage, recurrent colorectal cancer, distal metastasis, familial adenomatous polyposis (FAP), hereditary non-polyposis colorectal cancer (HNPCC), local resection, clinical T0-3 disease, or neoadjuvant chemoradiotherapy were excluded.

The charts of 293 consecutive patients who had undergone curative surgery for colorectal cancer with a perforated tumor and/or suspected involvement of another organ (T4) diagnosed by computed tomography (CT) and/or magnetic resonance imaging (MRI) were reviewed. Of these patients, 71 (24.2 %) underwent laparoscopic colorectal resection, and 222 (75.8 %) underwent open resection.

Demographic data including age, sex, body mass index (in kilograms per meter squared, BMI), ASA (American Society of Anesthesiologists) score, preoperative carcinoembryonic antigen (CEA) level, previous abdominal operation history, tumor location, clinical T or N category, cancer obstruction or perforation, pathologic features (proximal and distal resection margin, the seventh American Joint Committee on Cancer (AJCC) TNM tumor stage, lymphatic invasion, vascular invasion, and perineural invasion), and adjuvant chemotherapy were collected and analyzed. In addition, we also collected and analyzed data on perioperative clinical outcomes (operative time; blood loss; days to first flatus; days to first diet; length of hospital stay; diverting stoma; and postoperative adverse events including anastomotic leakage, type of laparoscopic surgery, and open conversion rate). Clinical factors related to pathologic T4 stage were analyzed for the whole cohort and clinicopathologic factors for survival were also evaluated. All colorectal resections were performed with curative intent. This study was approved by the Institutional Review Board of Samsung Medical Center, Sungkyunkwan University.

All patients underwent physical examination, measurement of serum CEA level, colonoscopy, chest CT, and abdominopelvic CT or pelvic MRI for preoperative clinical staging. Positron emission tomography (PET) scanning was used to assess the extent of lymph node metastasis and distal metastasis, if indicated. T4 disease was preoperatively suspected in cases with a perforated tumor and/or invasion of adjacent organs on the above-mentioned preoperative imaging studies. Patients were followed-up at 3-month intervals for 2 years, at 6-month intervals for the next 3 years, and annually thereafter. On a semiannual basis or when there was suspicion of recurrence, follow-up examinations including a clinical history, physical examination, serum CEA assay, chest CT, abdominopelvic CT or MRI, colonoscopy, or PET scanning were performed. Recurrence was determined by clinical and radiological examination or by histologic confirmation. The main pattern of recurrence was recorded as the first site of detectable failure during the follow-up period.

Statistical analyses were carried out using the Statistical Package for the Social Sciences for Windows, version 18.0 (SPSS, Chicago, IL, USA). The significance of differences between groups was evaluated using the Chi-square test or analysis of variance, as appropriate. Survival rates were calculated using the Kaplan-Meier method, and prognostic factors and survival curves were compared using log-rank tests. Factors that were significant (p ≤ 0.1) upon univariate analysis were entered into multivariate analysis using the Cox model. A *P*-value of 0.050 or less was considered statistically significant.

## Results

The demographic features of the open and laparoscopic groups are presented in Table 1. There were no differences in age, sex, ASA score, previous operative history, tumor location, or clinical N stage. BMI was higher in the laparoscopic resection group than in the open surgery group (*p* = 0.011). In addition, preoperative CEA level, combined resection of an adjacent organ, proportion of clinical T4b, cancer obstruction, and perforation were significantly higher in the open surgery group compared to the laparoscopic surgery group (Table 1). The median follow-up period was 36.0 months (range, 0 – 121 months).

**Table 1:** Demographic features of the study group

	Open surgery	Laparoscopic surgery	*P*-value
	*n* = 222	*n* = 71	
Age (years)			0.321
median (range)	61.0 (17–84)	59.0 (36–80)	
Sex			0.372
Female	96 (43.2 %)	35 (49.3 %)	
Male	126 (56.8 %)	36 (50.7 %)	
BMI (kg/m^2^)			0.011
median (range)	22.6 (15.2-31.3)	23.6 (17.3-31.9)	
ASA score			0.377
1	90 (40.5 %)	33 (46.5 %)	
2 + 3 + 4	132 (59.5 %)	38 (53.5 %)	
Preoperative CEA (ng/ml)			0.005
mean ± SD	9.5 ± 20.6	4.5 ± 8.6	
Previous abdominal operation history			0.169
Negative	48 (21.6 %)	21 (29.6 %)	
Positive			
Combined resection of an adjacent organ (%)	73 (32.9 %)	7 (9.9 %)	<0.001
Location of tumor (%)			0.956
Right colon^a^	77 (34.70 %)	26 (35.60 %)	
Left colon^a^	77 (34.70 %)	24 (33.80 %)	
Rectum	68 (30.60 %)	21 (29.60 %)	
Clinical T4 status			<0.001
T4a	130 (58.6 %)	58 (81.7 %)	
T4b	92 (41.4 %)	13 (18.3 %)	
Clinical N status			0.523
Negative	9 (4.10 %)	4 (5.60 %)	
Positive	213 (95.90 %)	67 (94.40 %)	
Cancer obstruction (%)	90 (40.5 %)	17 (23.9 %)	0.011
Cancer perforation (%)	23 (10.4 %)	2 (2.8 %)	0.048

*CEA* Carcinoembryonic antigen

^a^The right colon and left colon were divided by the splenic flexure

When comparing pathologic outcomes, pathologic T stage, tumor size, and the number of harvested lymph nodes were significantly higher in the open surgery group. In addition, pathologic N stage and TNM stage in the laparoscopic surgery group were higher than those in the open group, although these differences were not statistically significant. Analysis of perioperative clinical outcomes showed no differences in operative time, diverting stoma, or clinical anastomotic leakage between groups. However, blood loss (175.0 ml versus 100.0 ml, *p* < 0.001) and overall postoperative adverse events (31.5 % versus 14.1 %, *p* = 0.004) in the open surgery group were higher than those in the laparoscopic surgery group. In addition, the number of days to first flatus (4.0 days versus 3.0 days, *p* = 0.001), days to first diet (5.0 days versus 4.0 days, *p* = 0.008), and length of hospital stay (12.0 days versus 9.0 days, *p* < 0.001) were shorter in the laparoscopic surgery group. The types of laparoscopic surgery modalities were as follows: 59.1 % (42 patients) underwent hand-assisted laparoscopic surgery, 31.0 % (22 patients) had conventional laparoscopic surgery, and 9.9 % (7 patients) had a single-incisional laparoscopic surgery. Postoperative adverse events occurred in 70 patients (31.5 %) in the open group, but in only 10 patients (14.1 %) in the laparoscopic group (*p* = 0.004). The primary adverse events were ileus (9.9 (22/222) in open surgery and 2.8 % (2/71) in laparoscopic surgery) and wound seroma (5.4 % (12/222) versus 4.2 % (3/71), respectively). Cases of anastomotic leakage did not differ significantly between the two groups, with nine patients (4.1 %) experiencing leakage in the open group versus one patient (1.4 %) in the laparoscopic group (*p* = 0.285). Notably, four (5.6 %) patients underwent open conversion during laparoscopic surgery; pneumoperitoneum could not be sustained during the operation for one patient, a suspected metastatic lymph node beyond the surgical plane was identified during the laparoscopic approach with severe adhesion in another patient, the surgical field could not be secured because of severe bowel edema due to partial obstruction of cancer in another patient, and localized abscess due to cancer perforation with severe adhesion was found in another patient (Table 2).

**Table 2:** Comparison of pathologic outcomes and perioperative clinical outcomes between the open and laparoscopic surgery groups

		Open surgery	Laparoscopic surgery	*P*-value
		*n* = 222	*n* = 71	
Perioperative features				
Type of surgery				0.296
Right hemicolectomy		69 (31.1 %)	21 (29.6 %)	
Transverse colectomy		4 (1.8 %)	0 (0.0 %)	
Left hemicolectomy		19 (8.6 %)	6 (8.5 %)	
Anterior resection		60 (27.0 %)	24 (33.8 %)	
Low anterior resection		52 (23.4 %)	20 (28.2 %)	
Hartmann’s operation		9 (4.1 %)	0 (0.0 %)	
Abdominoperineal resection		8 (3.6 %)	0 (0.0 %)	
Total colectomy		1 (0.5 %)	0 (0.0 %)	
Operation time (minutes), median (range)		155.5 (48–708)	155.0 (79–399)	0.249
Blood loss (ml), median (range)		175.0 (20–4200)	100.0 (20–450)	<0.001
Days to first flatus (days), median (range)		4.0 (1–67)	3.0 (1–11)	0.001
Days to first solid food (days), median (range)		5.0 (2–69)	4.0 (3–21)	0.008
Length of hospital stay (days), median (range)		12.0 (2–116)	9 (7–27)	<0.001
Diverting stoma		8 (3.60 %)	2 (2.8 %)	0.751
Pathologic outcomes				
Pathologic T stage no. ( %)	T2	9 (4.0 %)	3 (4.2 %)	0.041
	T3	120 (54.1 %)	50 (70.4 %)	
	T4	93 (41.9 %)	18 (25.4 %)	
Pathologic N stage no. ( %)	0	118 (53.2 %)	28 (39.4 %)	0.085
	1	57 (25.7 %)	27 (38.0 %)	
	2	47 (21.2 %)	16 (22.5 %)	
Tumor size (cm), median (range)		7.0 (1–20)	5.5 (2–12)	<0.001
Grade of differentiation no. ( %)				0.136
WD + MD		180 (81.1 %)	63 (88.7 %)	
PD + MUC + Signet		42 (18.9 %)	8 (11.3 %)	
Lymphatic invasion	Negative	143 (64.3 %)^a^	44 (62.0 %)	0.427
	Positive	70 (31.5 %)	27 (38.0 %)	
Vascular invasion	Negative	173 (77.9 %)^b^	62 (87.3 %)	0.914
	Positive	24 (10.8 %)	9 (12.7 %)	
Perineural invasion	Negative	176 (79.3 %)^c^	56 (78.9 %)	0.196
	Positive	30 (13.5 %)	15 (21.1 %)	
Harvested lymph nodes, median (range)		25.0 (4–138)	20.0 (7–52)	<0.001
Proximal resection margin, median (range)		11.0 (2–57)	7.0 (3–28)	<0.001
Distal resection margin, median (range)		5.5 (0–55)	4.5 (1–28)	0.241
Adjuvant chemotherapy		171 (77.0 %)	60 (84.5 %)	0.179
Recurrence		35 (15.8 %)	10 (14.1 %)	0.732
Local recurrence		11 (5.0 %)	2 (2.8 %)	0.741
Distant recurrence		24 (10.8 %)	8 (11.3 %)	0.914
Postoperative complications				
Overall		70 (31.5 %)	10 (14.1 %)	0.004
Wound seroma		12 (17.1 %)	3 (30 %)	
Wound dehiscence		7 (10.0 %)	0 (0 %)	
Paralytic ileus		22 (31.4 %)	2 (20 %)	
Mechanical obstruction		2 (2.9 %)	0 (0 %)	
Urinary retention		7 (10.0 %)	2 (20 %)	
Intraabdominal abscess		2 (2.9 %)	0 (0 %)	
Intraabdominal bleeding		1 (1.4 %)	1 (10 %)	
Gastrointestinal bleeding		1 (1.4 %)	0 (0 %)	
Chylous ascites		2 (2.9 %)	0 (0 %)	
Urinary injury		0 (0.0 %)	1 (10 %)	
Pneumothorax		1 (1.4 %)	0 (0 %)	
Acute myocardial infarction		1 (1.4 %)	0 (0 %)	
Pneumonia		2 (2.9 %)	0 (0 %)	
Delirium		1 (1.4 %)	0 (0 %)	
Anastomotic leakage		9 (12.9 %)	1 (10 %)	0.285
HALS/LAP/SILS			42 (59.1 %)	
			22 (31.0 %)	
			7 (9.9 %)	
Open Conversion			4 (5.6 %)	

*WD* Well differentiated, *MD* Moderately differentiated, *PD* Poorly differentiated, *MUC* Mucinous adenocarcinoma, *signet* Signet ring cell type, *N/A* Not assessed, *HALS* Hand-assisted laparoscopic surgery, *LAP* Conventional laparoscopic surgery, *SILS* Single-incisional laparoscopic surgery

^a^ N/A 9 (4.2 %) cases

^b^ 25 (11.3 %) cases

^c^ 30 (13.5 %) cases

**Fig. 1:**
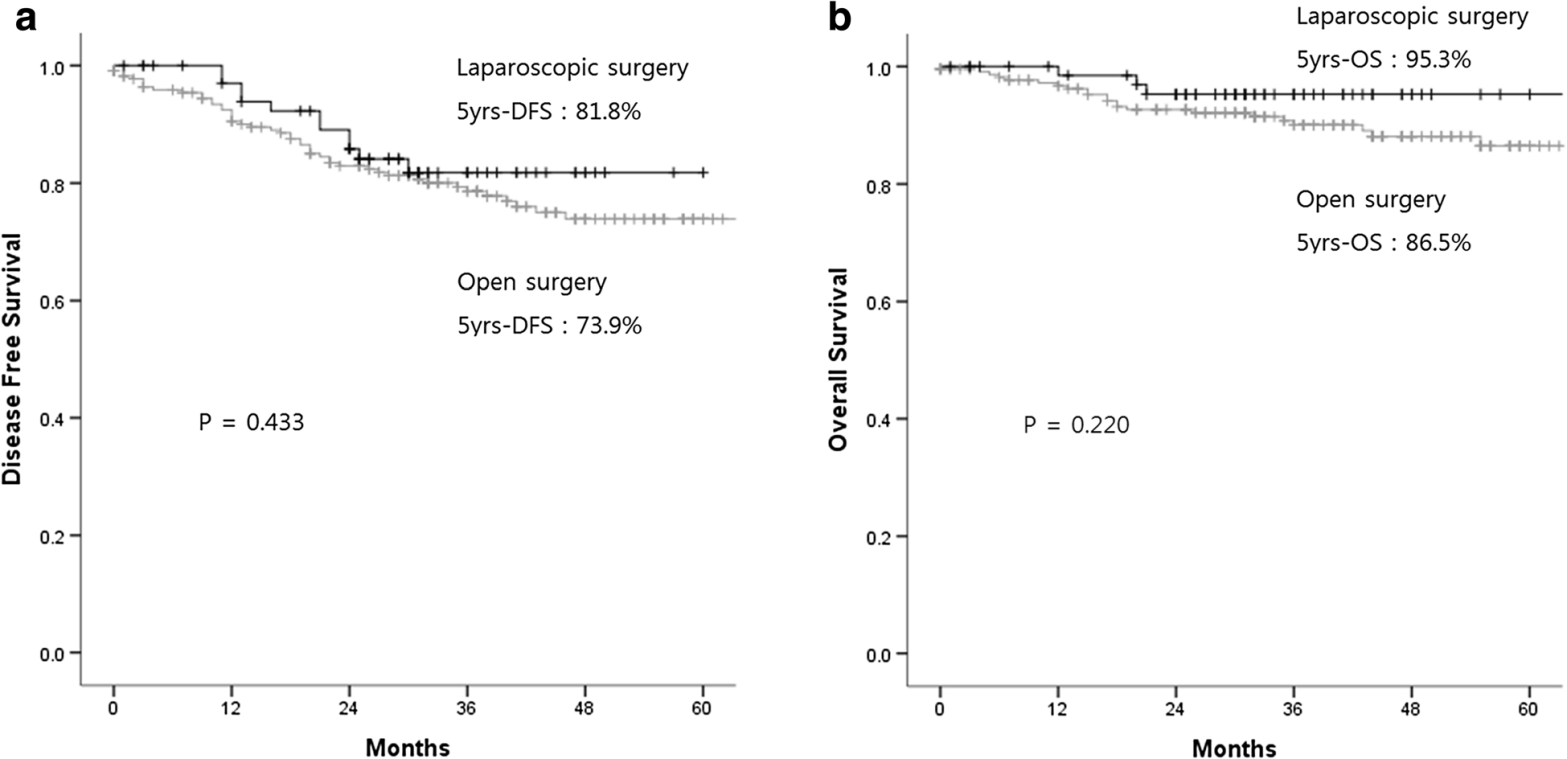
Disease-free survival rate and overall survival rate of patients who received laparoscopic versus open surgery. **a**: Disease Free Survival, **b**: Overall Survival

According to multivariate analysis, the single clinically predictive factor of pathologic T4 staging was clinically suspicious perforation (*p* = 0.024) (Table 3). Multivariate analysis also showed the strongest independent prognostic factors predicting lower disease-free survival to be age (>60) (*p* = 0.036), preoperative CEA level (>5 ng/ml) (*p* = 0.032), tumor location (rectum) (*p* < 0.001), and pathologically confirmed T4 staging (*p* = 0.006). Operative technique was not found to affect prognosis (Table 4). In addition, 5-year disease free survival (DFS) and 5-year overall survival (OS) in the laparoscopic surgery group were not statistically different from those of the open surgery group (81.8 versus 73.9 (*p* = 0.433), and 95.3 % versus 86.5 % (*p* = 0.220), respectively) (Fig. 1).

**Table 3:** Predictive factors for pT4 according to univariate and multivariate analyses

	Univariate analysis			Multivariate analysis		
	HR	95 % CI	*P*-value	HR	95 % CI	*P*-value
Age (years)						
>60 / ≤ 60	0.84	0.52-1.35	0.466			
Sex						
Male / Female	1.38	0.85-2.23	0.189			
BMI (kg/m^2^)						
>23 / ≤ 23	0.82	0.51-1.32	0.423			
PreOP CEA (ng/ml)						
>5 / ≤ 5	1.85	1.09-3.15	0.022	1.71	0.99-2.94	0.053
Tumor location						
Rectum / Colon	0.77	0.46-1.29	0.316			
Clinical T4 status						
T4b / T4a	1.56	0.96-2.54	0.076	1.43	0.75-2.43	0.179
Clinical N status						
+ / -	2.09	0.56-7.75	0.272			
Clinical obstruction						
+ / -	1.62	0.99-2.63	0.054	1.43	0.85-2.41	0.181
Clinical perforation						
+ / -	3.22	1.37-7.56	0.007	2.77	1.15-6.67	0.024
Operative technique						
Laparoscopic / Open	0.47	0.26-0.85	0.013	0.55	0.30-1.02	0.060

*PreOP CEA* Preoperative carcinoembryonic antigen

**Table 4:** Predictive factors for DFS according to univariate and multivariate analyses

	Univariate analysis			Multivariate analysis		
	HR	95 % CI	*P*-value	HR	95 % CI	*P*-value
Age (years)						
>60 / ≤ 60	1.79	1.06-3.02	0.028	1.86	1.04-3.31	0.036
Sex						
Male / Female	1.11	0.66-1.84	0.702			
BMI (kg/m^2^)						
>23 / ≤ 23	1.05	0.64-1.75	0.839			
PreOP CEA (ng/ml)						
>5 / ≤ 5	2.28	1.34-3.86	0.002	1.87	1.06-3.30	0.032
Previous abdominal operation						
+ / -	1.05	0.59-1.89	0.863			
Combined resection of an adjacent organ						
+ / -	1.40	0.82-2.38	0.217			
Tumor location						
Rectum / Colon	2.92	1.76-4.87	<0.001	3.25	1.85-5.74	<0.001
Clinical Obstruction						
+ / -	1.14	0.67-1.91	0.634			
Clinical Perforation						
+ / -	1.73	0.82-3.63	0.152			
Operative technique						
Laparoscopic / Open	0.77	0.40-1.49	0.435			
Pathologic T stage						
T4 / T2 + T3	1.77	1.07-2.95	0.027	2.22	1.26-3.93	0.006
Pathologic N stage						
+ / -	2.20	1.29-3.77	0.004	1.22	0.63-2.36	0.556
Differentiation of cell						
PD + MUC + Signet / WD + MD	1.35	0.73-2.50	0.333			
Lymphatic invasion						
+ / -	1.37	0.80-2.33	0.253			
Vascular invasion						
+ / -	2.08	1.15-3.75	0.015	1.60	0.79-3.26	0.194
Perineural invasion						
+ / -	1.79	0.90-3.56	0.097	0.81	0.38-1.76	0.600
Adjuvant chemotherapy						
+ / -	0.65	0.35-1.20	0.169			

*PreOP CEA* Preoperative carcinoembryonic antigen, *WD* Well differentiated, *MD* Moderately differentiated, *PD* Poorly differentiated, *MUC* Mucinous adenocarcinoma, *signet* Signet ring cell type

## Discussion

A total of 293 consecutive patients with clinically suspicious T4 cancer were enrolled in this study, and 5-year survival and perioperative outcomes were analyzed. The perioperative clinical outcomes of laparoscopic resection of clinically suspicious T4 colorectal cancer were more favorable than those of open surgery, with a smaller amount of blood loss, shorter hospital stay, and fewer postoperative adverse events. There were no differences in operative time between the two groups, and the rate of conversion to open from laparoscopic surgery was only 5.4 % (4/71). Age, preoperative CEA level, rectal location, clinical obstruction and pathologic T stage were associated with DFS, but operative technique did not affect prognosis. In addition, 5-year DFS and OS rates were not different between the two groups.

Several studies exploring the short-term outcomes of laparoscopic versus open approaches analyzed patients with pathologically established T4 disease [12, 14]. In these studies, it was reported that laparoscopic treatment of T4 colon cancer was safe and feasible and provided similar surgical and oncological outcomes to the open technique. However, these studies also showed that it is difficult to determine the approach modality, open or laparoscopic, in patients with confirmed pathologic T4 disease. In actual clinical settings, the modality is determined preoperatively based on the results of preoperative CT or MRI imaging. Thus, studies exploring the choice between laparoscopic and open approaches based on clinical factors in suspected T4 colorectal cancer are needed. Some studies exploring this issue have been reported, but the power of these studies is lacking due to small sample sizes [8, 11].

In this study, several factors influenced the preoperative decision regarding type of approach. Analysis of demographic features between the two groups revealed that these factors included clinical suspicion of T4b disease, cancer obstruction, and cancer perforation. Clinically suspected T4b disease compared to T4a disease, cancer obstruction, and perforation were significantly more prevalent in the open group than in the laparoscopic group. On the other hand, postoperative clinical N stage did not differ between groups.

Patients have been undergoing laparoscopic surgery since 2007 when laparoscopic surgery was first performed in a cohort of suspected clinical T4 colon cancer patients (data not shown). At that time, only one laparoscopic colectomy (6.25 %) was performed. Since then, the proportion of laparoscopic surgeries has increased gradually up to 41.5 % (39/94) in 2010. Since 2000, when laparoscopic colectomy first became available for cancer patients at our institution, the indications for laparoscopic procedures have expanded with growing surgeon experience [15]. According to our results, the open conversion rate has not changed over time because all four open conversion cases occurred in 2009. The only clinical factor found to be significantly predictive of pathologic T4 disease was clinical cancer perforation; preoperative CEA level showed a trend toward statistical significance (*p* = 0.053). It would be too hard to predict pathologic T stage by clinical factors alone. One study reported that the presence of a T4 tumor was a risk factor for conversion, and conversion to an open approach during laparoscopic rectal resection was associated with increased postoperative morbidity [16]. In cases of suspected T4 disease, the choice of approach, laparoscopic or open, should be made prudently in order to obtain proper resection margins and to offer better prognosis. However, the conversion rate could be minimized with growing surgeon experience. We experienced four (5.6 %) open conversion cases, and this rate was considered acceptable in patients with locally-advanced colorectal cancer.

Although there were differences in the number of harvested lymph nodes between the two groups, the median number of lymph nodes in the laparoscopic group was 20, and the number of patients with less than 12 harvested lymph nodes, as suggested in the National Comprehensive Cancer Network (NCCN) guidelines [17], was only 24 (8.2 %) (17 (7.7 %) in the open group and seven (9.9 %) in the laparoscopic group, *p* = 0.556). Despite this, there were no differences between the two groups in disease-free survival, distant metastasis, or local recurrence. Moreover, operative technique was not a significant prognostic factor for disease free survival in multivariate analysis.

Limitations of this study included clinicopathologic differences between two groups, its single-institution, retrospective nature, and the small sample size. However, our results are meaningful despite the potential selection bias because it is difficult to design a study based on retrospective data. Well-designed prospective studies are needed to confirm our findings. An additional limitation is that the indications for surgical approach were unclear. There appeared to be a trend in which patients for whom disease progression was suspected clinically were converted to open surgery from the laparoscopic approach because the indications for laparoscopic surgery were not clearly established. As mentioned before, Park et al. [15] reported that the indications for laparoscopic surgery have expanded with accumulating experience of surgeon. The laparoscopic approach is carefully considered for clinically suspected T4 colorectal cancer at our institution. We did not analyze the inter-rater variability between surgeons. Five surgeons were involved in our study, but only four surgeons performed more than 200 laparoscopic colorectal surgeries, with the fifth surgeon having performed 50 surgeries before first performing surgery for suspected T4 colorectal cancer. Randomized controlled trials regarding the surgical learning curve should be considered closely, even when experienced surgeons are involved. One of the surgeons performed only hand-assisted laparoscopic surgery. Patients who underwent neoadjuvant chemoradiotherapy were excluded in this study because the pathology and surgical circumference in the operative field would be different pre- and post-neoadjuvant chemoradiotherapy [18]. Finally, the accuracy of the preoperative staging of colorectal cancer was reported to range from 47.5 to 80 % [19]. However, the positive predictive value for clinical T4 disease might be as low as 19.4 to 51.2 % [11, 20]. The positive predictive value in this study was 37.9 % (111/293). A relatively large number of patients who were clinically suspected of having T4 disease were pathologically shown to have T3 disease. The accuracy of predicting T stage via preoperative imaging modalities might be another limitation of this study.

## Conclusions

In conclusion, despite the clinical suspicion of T4 disease before surgery, laparoscopic colorectal resection for T4 colorectal cancer can be attempted and has similar perioperative and long-term oncologic outcomes to those of the open approach when performed by an experienced surgeon.

## Abbreviations

AJCC: American Joint Committee on Cancer
ASA: American Society of Anesthesiologists
ASCRS: American Society of Colon & Rectal Surgeons
BMI: Body mass index
CEA: Carcinoembryonic antigen
CT: Computed tomography
DFS: Disease free survival
FAP: Familial adenomatous polyposis
HNPCC: Hereditary non-polyposis colorectal cancer
MRI: Magnetic resonance imaging
NCCN: National Comprehensive Cancer Network
OS: Overall survival
PET: Positron emission tomography
SAGES: Society of American Gastrointestinal and Endoscopic Surgeons guidelines
SPSS: Statistical Package for the Social Sciences
